# Liposomes: from August Wassermann to vaccines against COVID-19

**DOI:** 10.5599/admet.1926

**Published:** 2023-06-30

**Authors:** Ganna Grygorieva, Daria Pylypenko, Yuriy Krasnopolsky

**Affiliations:** 1 SI “Institute of Pharmacology and Toxicology of NAMS of Ukraine”, 14, Ezhene Potier Str., Kyiv, 03680, Ukraine; 2 State Biotechnological University, 44, Alchevskikh str., Kharkiv, 61002, Ukraine; 3 National Technical University “Kharkiv Polytechnic Institute”, 2, Kyrpychova str., Kharkiv, 61002, Ukraine

**Keywords:** liposomal antigen delivery system, cardiolipin antigen, antilipid antibodies, Wassermann reaction, serodiagnosis of syphilis, lipid nanoparticle, mRNA vaccine

## Abstract

**Background and Purpose:**

The development of vaccines against the SARS-CoV-2 virus has become a big challenge for many countries in 2020-2022. mRNA vaccines were shown to be effective and safe and have been widely used worldwide in the fight against the COVID-19 pandemic. The fundamental factor in creating mRNA vaccines, which ensures effective delivery of mRNA to the host cells, is the composition of lipid nanoparticles, namely the presence of ionized charged lipids, which ensures the binding of mRNA molecules. However, the significant role of liposomes in the development of liposomal vaccines and identification of immunochemical reactions involving lipids should be assessed in the context of the development of the pioneering idea of August Wassermann about the use of liposomal antigens in the diagnosis and immunoprophylaxis of serious human diseases.

**Experimental Approach:**

The review is devoted to the use of liposomal antigens as antigen-delivery systems for diagnosis and immunoprophylaxis.

**Key Results:**

Studies of cardiolipin antigen in serodiagnosis of syphilis became the foundation of antibodies in diagnosing various infectious diseases and pathological conditions, such as tuberculosis, lupus erythematosus, COVID-19, borreliosis, etc. Identification of antiphospholipid antibodies (mainly anticardiolipin) and today is the most important diagnostic tool for antiphospholipid syndrome.

**Conclusion:**

The liposomal system first proposed in 1906 for the diagnosis of syphilis evolved more than a century later into mRNA vaccines, which are used today in the fight against the COVID-19 pandemic.

## Introduction

**Figure 1.**
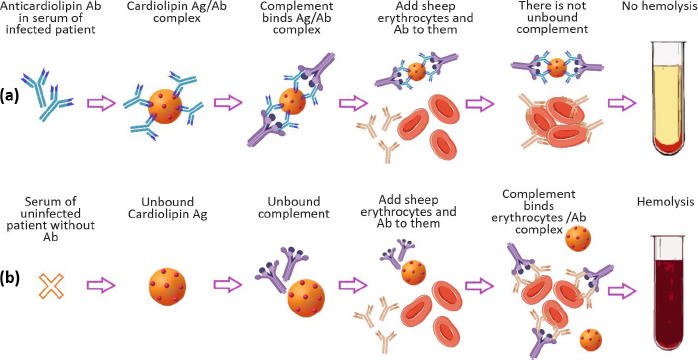
Principle of Wassermann reaction: **(a)** positive result and **(b)** negative result.

In modern concepts of drug delivery systems, a priority place is occupied by nanosized lipid structures, which for the last 40 years, have become the applied basis for liposomal drug and antigen delivery systems for pharmacotherapy, diagnostics, and immunoprophylaxis [1,2]. However, it should be recalled that the successful empirical use of lipid nanoparticles in laboratory diagnostics of infectious diseases dates back to the beginning of the 20th century. We are talking about the first serological test proposed in 1906 by A. Wassermann, A. Neisser and С. Bruck for the diagnosis of *Treponema pallidum* (syphilis) in humans using the complement fixation reaction with lipid antigens (Ag), called “Wassermann reaction” (Figure 1) [3].

Despite the development of methods for obtaining various lipid Ags and modification of reactions involving these Ags in subsequent years, only cardiolipin (diphosphatidylglycerol – DPG) was identified as an immunochemically active lipid Ag for the serodiagnosis of syphilis fabrics due to its presence in both healthy and syphilitic tissues [4,5]. In addition to DPG itself, a composition of DPG and phosphatidylcholine (PC) was proposed as a lipid Ag for syphilis serodiagnosis, the components of which were isolated from the heart muscle of cattle. Note that in the second half of the 20^th^ century, the unsatisfactory specificity of these Ags in serodiagnosis initiated the study of a whole spectrum of cardiolipin Ags (so-called VDRL-test), differing in qualitative and quantitative composition [6-8].

## Liposomal structure of the cardiolipin antigen in the serodiagnosis of syphilis

**Figure 2.**
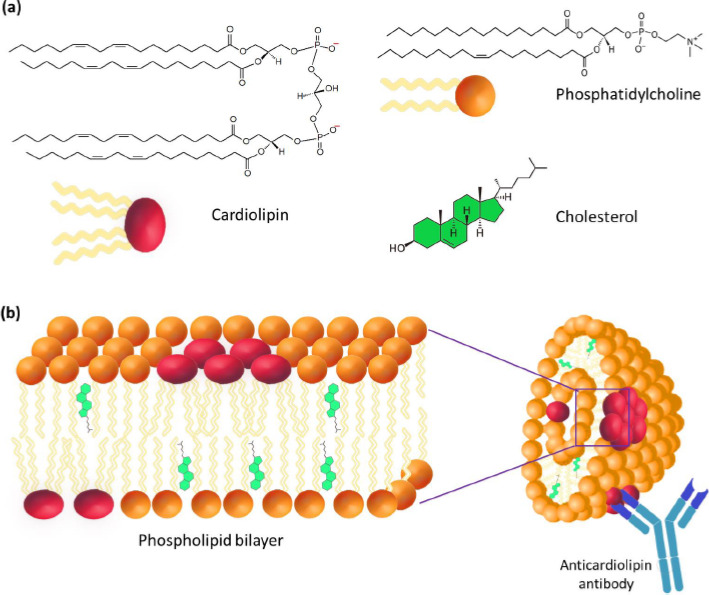
**(a)** Structure of cardiolipin, PC, and Chol. **(b)** Liposomal structure of the cardiolipin antigen based on cardiolipin, PC, and Chol.

The most important step in the study of cardiolipin Ag was the confirmation of its liposomal structure in the aqueous phase, which is no longer in doubt today. A number of studies have shown that the high immunochemical activity of cardiolipin Ag is a characteristic of the DPG liposomal composition. PC and cholesterol (Chol) with a particle size of 1 to 10 μm (Figure 2) [9-11]. Using VDRL-test based on DPG, PC, and Chol, the possibility of agglutination of antibodies with an emulsion of large bilayer liposomes containing not treponemal but lipid Ag was shown [12]. When the emulsion is mixed with serum, the antibodies in the serum of a patient with syphilis react specifically and with high affinity with DPG, causing agglutination.

A lipid composition including a negatively charged lipid (*e.g.* DPG, phosphatidylglycerol (PG), phosphatidylserine (PS) or phosphatidic acid (PA)), neutral lipid (*e.g.*, PC) and Chol was offered [13-15]. Using 31P-NMR, it was established that cardiolipin Ag is represented in the form of multilayer liposomes no less than 1 μm in size, consisting of a large number of bilayer phospholipid membranes [13-15]. The presence of phosphoglycerides (*e.g.*, phosphatidylethanolamine, lysophosphatidylcholine, and products of lipid peroxidation) in the antigen leads to the appearance in liposomes of regions with differences in the bilayer structure. The violation of the integrity of the bilayer membrane results in a distortion of the biological activity of Ag that decreases the specificity of serological reactions [16].

DPG is the only component of Ag capable of directly interacting with the active centers of antilipid antibodies [10,17]. In this case, PC and Chol are considered structural components of Ag, which allow the antigen-antibody complex to bind, complement or form a precipitate. Perhaps, PC forms a phospholipid bilayer where the antigenic determinants of DPG are located, and Chol provides rigidity to the lipid structure. The study of the ultrastructure of cardiopin Ag showed that its sensitivity and specificity were significantly reduced in the absence of Chol [18]. The study of the morphology of VDRL liposomes showed that most of Chol is located outside the lamellar membranes of PC and DPG, and its role is in the mechanical surface dispergation of DPG and PC [19]. Presumably, Chol does not affect the structure of epitopes that react with antibodies in the serum of syphilis patients.

## Antilipid antibodies in the infectious process

Lipid Ags used to diagnose syphilis should functionally detect antibodies that specifically bind to antigenic determinants on the surface of liposomal nanoparticles. Over the past 100 years, researchers in the field of immunology and lipidology have offered various explanations for the origin of antilipid antibodies in syphilis and other infectious diseases. The possibility of releasing lipid components from damaged treponema cells, which naturally leads to an autoimmune reaction, was discussed. The predictable phenomenon of induction of the so-called “non-treponemal" lipid antibodies in the infected organism in response to the *Treponema pallidum* lipid complex is also of interest. To confirm this hypothesis, the lipid composition of different strains of *Treponema pallidum* was studied by Reiter, Kazans, Nichols, and others [20-24]. In *Treponema pallidum*, DPG, PC, and Chol were identified as basic lipids, which content and ratio significantly depended on the nature of the strain, and the presence of varying amounts of sphingomyelin, phosphatidylethanolamine, phosphatidylinositol, and lysophosphatidylcholine was also determined. The average content of DPG in *Treponema pallidum* is estimated at 13-18 %, except for the Kazans strain, in which only trace amounts of DPG were determined at a relatively high content of PC (up to 40 %). Perhaps, the presence of the lipid complex DPG, PC, and Chol in the membrane of *Treponema pallidum* causes the appearance of antilipid antibodies specific to DPG in patients with syphilis. When studying the localization of cardiolipin Ag in treponema, it was found that lipids are present in the outer membrane of the causative agent of syphilis [25,26].

## Cardiolipin antigen in immunodiagnostics

During immunization of animals with compositions based on DPG or PI, PC, and Chol, antilipid antibodies in serum was detected using complement fixation reaction and microprecipitation reaction [27]. In this case, the immunogenicity of lipids is directly related to their liposomal organization. The possibility of obtaining antilipid antibodies against anionic phospholipids (DPG and PG) by long-term immunization of rabbits with cardiolipin Ag and Ag complexes with methylated BSA (bovine serum albumin) or cytochrome C has been shown [24,28]. Antibodies interacting with Ag in low titers are detected in the blood of animals that were immunized with individual lipids. The use of complexes of cardiolipin Ag with proteins causes a significant increase in titers of antilipid antibodies, most significant for cytochrome C [29]. The composition of antilipid antibodies interacting with DPG is mainly represented by G- and M-immunoglobulins, but IgA is also detected in severe stages of the disease [30].

The high titer of antilipid antibodies in syphilitic infection is the result of the combined action of both *Treponema pallidum* cardiolipin Ag and cellular DPG from damaged host tissues. Both factors can lead to the formation of anticardiolipin antibodies [20]. In recent years, this thesis has been confirmed not only for *Treponema pallidum*, but also for other infections [31]. Lipids play an irreplaceable structure-forming and functional role in various cellular processes, such as membrane fusion, division, endocytosis, transfer and functions of proteins, etc. At the same time, the influence of lipids on the development of the infectious process can be realized both as a result of interaction with the host membrane to facilitate the penetration of pathogens, and as a result of the support of certain functions of a pathogenic microorganism to increase its survival and proliferation. These features of the interaction with host lipids explain the appearance of antibodies against lipids, in particular, against phospholipids [32]. Using antiphospholipid ELISA, the immune response in the form of the production of antibodies against host phospholipids was studied in animals (mice) and humans [32]. Several phospholipids (namely PS, PA, PC) are targets for antibodies that occur at the early stage of infection, and a humoral immune response occurs to the same phospholipids in patients with acute infection and infected mice.

The use of the liposomal form of cardiolipin Ag, which has already become quite routine in complement fixation, agglutination, or microprecipitation reactions, has recently been enriched by the creation of new methodological approaches to the detection of antilipid antibodies [33]. An innovative platform for the detection of anticardiolipin antibodies in the serum of patients with syphilis was proposed using a self-assembled (3-mercaptopropyl)-trimethoxysilane monolayer for immobilization of an antigenic suspension containing DPG, PC, and Chol as a biorecognition element [34]. The biosensor platform with a detection limit of anticardiolipin antibodies at 1:1024 and high selectivity for nonspecific biomolecules was evaluated by cyclic voltammetry, spectroscopy, electrochemical impedance, and atomic force microscopy.

Most of the current research on the detection and quantification of antiphospholipid antibodies is focused on enzyme-linked immunosorbent assay (ELISA) or radioimmunoassay (RIA). The algorithm of ELISA is based on the inhibition of the activity of certain enzymes (namely peroxidase and glucose oxidase) due to the interaction of the DPG-enzyme complex with antibodies. The use of cardiolipin Ag in the detection of antilipid antibodies by ELISA was proposed [35].

Liposomal forms of antigens are used to diagnose infectious diseases and detect antibodies in various pathological conditions in which antilipid antibodies appear. Studies of Ag in serodiagnosis of syphilis actually became the foundation for using antilipid antibodies for the diagnosis of various infectious diseases and pathological conditions, such as tuberculosis [36,37], lupus erythematosus [38], COVID-19 [39], borreliosis [32], *etc.* [41,42]. Liposomal forms of antigens containing secretory proteins of mycobacteria are also proposed for the diagnosis of tuberculosis [43]. Liposomal antigens are used to study a number of allergic diseases in their diagnosis and obtain antibodies in model experiments [44]. Identification of antiphospholipid antibodies (mainly anticardiolipin) is the most important diagnostic tool for a serious disease called antiphospholipid syndrome [40,45]. In this case, the diagnosis is carried out using liposomal antigens, mainly with cardiolipin. Antiphospholipid antibodies correlate with a predisposition to arterial or venous thrombosis, fetal loss, or thrombopenia. However, levels of antilipid antibodies, particularly anticardiolipin antibodies, do not correlate well with disease activity or specific features such as arthritis or kidney damage. Nevertheless, the presence of antilipid antibodies serves as a marker of thromboembolic complications. For these studies, it is possible to use antigens in liposomal form [46]. Thrombosis-associated antibodies detected in primary and secondary antiphospholipid syndrome also appear in lupus, myocardial infarction in patients with neurological symptoms, showing a clear correlation with the extent of the disease. It is important to note that antibodies in antiphospholipid syndrome have a different specificity compared to antibodies that accompany common infections, such as syphilis, malaria, parasitic diseases, and infectious mononucleosis [44].

## Liposome in COVID-19 vaccine development

In the second half of the 20^th^ century the development of research in the field of biophysics and biochemistry of lipids not only introduced the concepts of "lipid nanoparticle" and "liposome" in relation to immunochemical reactions involving lipids [47,48] but also resulted in the development of a new direction in pharmacy and medicine associated with the design and use of a class of innovative liposomal drugs for diagnosis, pharmacotherapy and immunoprophylaxis [49-54].

In 2020-2021, the appearance of vaccines of various structures and compositions against the SARS-CoV-2 virus on the global pharmaceutical market became a milestone achievement in the field of liposomal technologies [55,56]. The high pace of vaccine development in the alarming conditions of the COVID-19 pandemic was predetermined by the purposeful combination of the results of applied research on a wide class of lipids with fundamental information about the properties of messenger RNA (mRNA).

mRNA is synthesized during DNA transcription, contains information about the primary structure of proteins, and is used during translation as a template for their subsequent synthesis [57]. mRNA technology provides an innovative platform for *in situ* antigen expression with the advantage that mRNA-based vaccines (unlike DNA-based) do not integrate into chromosomes, avoiding the risks of oncogenesis and insertional mutagenesis [58-60].

G. Grigoriadis was the first to formulate significant advantages of the liposomal organization of mRNA-based vaccines [61,62]. mRNA encapsulated in the liposome is completely protected from nuclease attack in the bloodstream and penetrates the cell cytoplasm by endocytosis. At the same time, mRNA in cationic liposomes escapes the lysosomotropic pathway and remains intact in the cytoplasm. In the cytoplasm, mRNA is expressed as a spike protein, after which liposomes or lipid fragments exhibit their immunological adjuvant effect according to unclear mechanisms. These factors provide the possibility of quantitative functional encapsulation of mRNA into liposomes and safe biodegradation of nanoparticles.

Encapsulation of mRNA into lipid nanoparticles (LNPs) in the development of vaccines ensures the capture of protected mRNA by host cells and its delivery into the cytosol, where the mRNA sequence is translated into S-protein in ribosomes [63]. After post-translational processing in host cells, the S protein as a membrane-bound antigen provides the target Ag for B cells. Intramuscular administration of mRNA vaccine based on LNPs leads to temporary punctate inflammation, which promotes the recruitment of neutrophils and antigen-presenting cells (APC) to the delivery site [64].

The mRNA part of the genetic code of the virus leads to the synthesis of antigenic protein structures, in response to which specific antibodies appear in the human body [65]. After the administration of mRNA during vaccination, viral spike proteins begin to be produced, and neutralizing antibodies against spike proteins and cellular immune responses can prevent infection.

The mRNA vaccine development platform based on lipid nanosystem was first used for COVID-19 vaccine creation by Pfizer-BioNTech and Moderna [66]. Unlike traditional vaccines, which can deliver an inactivated or attenuated version of the virus (such as a capsular protein), these vaccines deliver genetic information to the body, synthesizing a protein to generate an immune response. The mRNA vaccine against COVID-19 encodes the spike glycoprotein S spike protein of the SARS-CoV-2 virus, which is used by the virus to penetrate into human cells.

**Figure 3.**
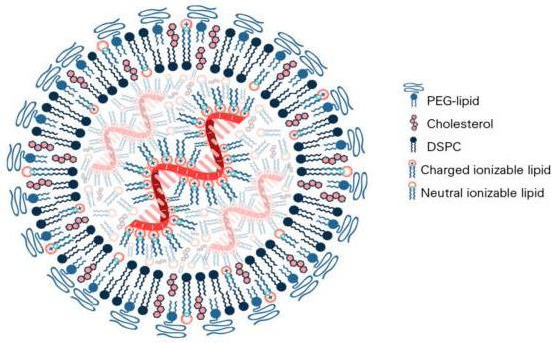
Structure of the LNP in mRNA vaccine [55]

In the composition of mRNA vaccines against COVID-19, the lipid component is represented by several lipids, among which so-called "ionized" lipid is functionally accentuated, and the rest stabilize the structure of LNPs and provide the stability of the vaccine in the body (Figure 3) [55].

Similarly to classical liposomes [2], the cationic charge of the main lipid contributes to obtaining the optimal structure of LNPs. The Pfizer-BioNTech COVID-19 vaccine contains four lipid components [67,68]: 50 % ionized lipid ((4-hydroxybutyl)azandiyl)-bis(hexane-6,1-diyl)-bis(2-hexyl-decanoate)), 10 % DSPC, 38.5 % Chol and 1.5 % PEG lipid ([(polyethylene glycol)-2000]-N,N-ditradecylacetamide). The LNP size ranges from 100 to 170 nm.

The algorithm for polylipid composition design of mRNA vaccine aims to form LNP and encapsulate the mRNA into its structure due to the interaction of the anionic phosphate backbone of the mRNA with the ionized lipid. In the design process, the mRNA contacts four lipids in a water-organic environment under the control of pH level and hydrophobic-hydrophilic balance to ensure the binding of the mRNA to the ionized lipid. Bound mRNA is identified in the inner cavity of multilayer LNPs formed by complementary lipid components. In this case, DSPC forms the main bilayer of the liposomal membrane, and the presence of PEG-lipid and Chol ensures optimal hydrophilicity, stability, and size of LNPs with encapsulated mRNA.

A liposome-based vaccine candidate EG-COVID differs from the composition of Pfizer-BioNTech product, consists of DOTAP (1,2-dioleoyl-3-trimethylammonium-propane), DOPE (1,2-dioleoyl-sn-glycero-3-phosphoethanolamine) and Chol, induces stable humoral and cellular immunity to the SARS-CoV-2 virus, and suppresses the SARS-CoV-2 viral infection in Vero cells [69]. The optimization of the LNP lipid spectrum of the composition of the lyophilized form of EG-COVID can increase compliance in the standardization of the product and the process of immunoprophylaxis.

## Conclusions

In conclusion, the significant role of liposomes in the development of liposomal vaccines against COVID-19 and the identification of immunochemical reactions involving lipids should be assessed in the context of the development of the pioneering idea of August Wassermann about the diagnostic use of liposomal antigens in the creation of innovative liposomal preparations for the diagnosis and immunoprophylaxis of serious human diseases. Thus, the liposomal system first proposed in 1906 for the diagnosis of syphilis (Figure 2) evolved more than a century later into mRNA vaccines (Figure 3), which are used today in the fight against the COVID-19 pandemic.


**Conflict of interest** *: The author declares no conflict of interest.*
